# Death of Dr. John T. Shotwell

**Published:** 1850-08

**Authors:** 


We regret to learn the death of Dr. John T. Shotwell, Professor of Anatomy in the Medical College of Ohio. He is said to have died of Cholera.

